# Diversity in sphingolipid metabolism across land plants

**DOI:** 10.1093/jxb/erab558

**Published:** 2022-02-03

**Authors:** Tegan M Haslam, Ivo Feussner

**Affiliations:** 1 University of Goettingen, Albrecht-von-Haller-Institute for Plant Sciences, Department of Plant Biochemistry, Justus-von-Liebig-Weg 11, D-37077, Goettingen, Germany; 2 University of Goettingen, Goettingen Center for Molecular Biosciences (GZMB), Service Unit for Metabolomics and Lipidomics, Goettingen, Germany; 3 University of Goettingen, Goettingen Center for Molecular Biosciences (GZMB), Department of Plant Biochemistry, Goettingen, Germany; 4 Academia Sinica, Taiwan

**Keywords:** *Arabidopsis thaliana*, ceramide, desaturase, glycosyl inositol phosphorylceramide (GIPC), metabolic diversity, *Oryza sativa*, *Physcomitrium patens*, sphingolipid

## Abstract

Sphingolipids are essential metabolites found in all plant species. They are required for plasma membrane integrity, tolerance of and responses to biotic and abiotic stresses, and intracellular signalling. There is extensive diversity in the sphingolipid content of different plant species, and in the identities and roles of enzymes required for their processing. In this review, we survey results obtained from investigations of the classical genetic model *Arabidopsis thaliana*, from assorted dicots with less extensive genetic toolkits, from the model monocot *Oryza sativa*, and finally from the model bryophyte *Physcomitrium patens*. For each species or group, we first broadly summarize what is known about sphingolipid content. We then discuss the most insightful and puzzling features of modifications to the hydrophobic ceramides, and to the polar headgroups of complex sphingolipids. Altogether, these data can serve as a framework for our knowledge of sphingolipid metabolism across the plant kingdom. This chemical and metabolic heterogeneity underpins equally diverse functions. With greater availability of different tools for analytical measurements and genetic manipulation, our field is entering an exciting phase of expanding our knowledge of the biological functions of this persistently cryptic class of lipids.

## Teaser

Sphingolipid profiles vary substantially across terrestrial plant lineages. This review describes those of several model systems, and what we can infer about sphingolipid biosynthesis and physiological function from this metabolic diversity.

## Introduction

Sphingolipids are essential metabolites found in all eukaryotic cells. They have superficial structural similarity to glycerolipids, the more common components of biological membranes, but have distinct chemistry, metabolism, and functions. Their unusual nature was the reason for their ‘sphingo-’ denomination; in 1884, the biochemist and physician Johann L.W. Thudichum used this term to describe these components of brain tissue, which he found confounding, in reference to the riddle of the sphinx in ancient Greek mythology (Thudichum, 1884). The scope of our understanding of sphingolipids is broader today, but arguably no less puzzling.

Across kingdoms, sphingolipids are essential for plasma membrane integrity, signalling, cell polarity and polar secretion, programmed cell death, and cellular responses to both biotic and abiotic factors in the environment. Their functions can also be highly specialized in different lineages. They have been extensively studied in animal systems due to their multiple, essential functions in the nervous system, and diseases associated with defects in sphingolipid metabolism (reviewed in Hannun and Obeid, 2018). Sphingolipid functions are equally essential for plant life, yet our understanding here is comparatively vague. Nevertheless, recent improvements in analytical methods for detecting and quantifying sphingolipids (Markham *et al.*, 2006; Markham and Jaworski, 2007; Buré *et al*., 2011, 2014, 2016; Cacas *et al*., 2012, 2013, 2016; Tarazona *et al.*, 2015; Ishikawa *et al.*, 2016), as well as genome editing technologies available in different plant species (reviewed in Hahne *et al.*, 2019), will greatly facilitate research. A clearer picture of both the biochemical and functional diversity of plant sphingolipids is currently emerging.

**Fig. 1.**
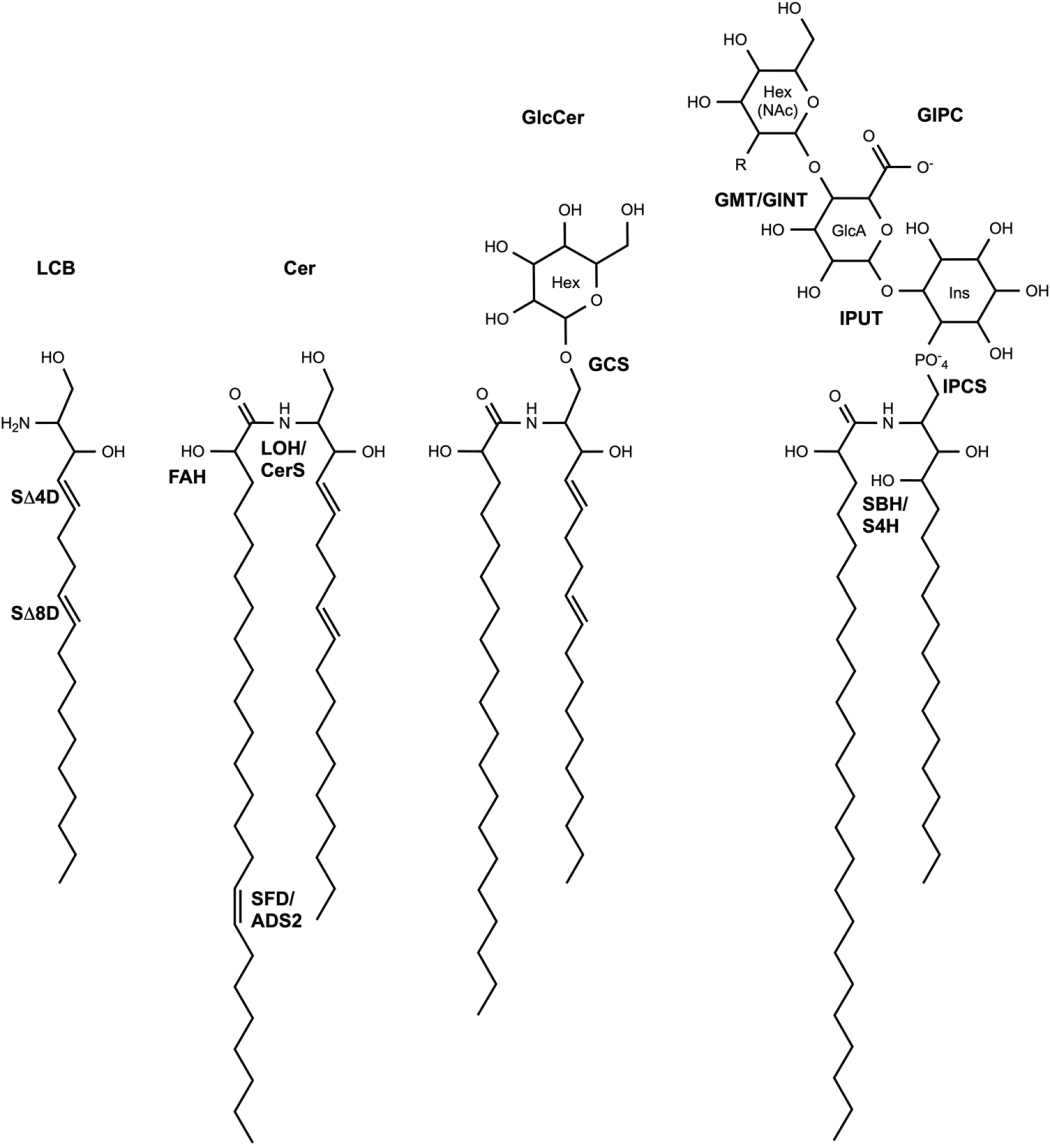
Representative structures of a free long chain base (LCB), ceramide (Cer), glucosylceramide (GlcCer), and glycosyl inositol phosphorylceramide (GIPC). Enzymes responsible for modification or addition of moieties are indicated in bold. DES/SΔ4D, degenerative spermatocyte/sphingolipid Δ4 desaturase/dihydroceramide desaturase; SLD/SΔ8D, sphingolipid Δ8 desaturase; LOH/CerS, longevity-assurance gene one homologue/ceramide synthase; FAH, fatty acyl hydroxylase; ADS/SFD, acyl-CoenzymeA desaturase/sphingolipid fatty acyl desaturase; GCS, glucosylceramide synthase; IPCS, inositol phosphorylceramide synthase; IPUT, inositol phosphorylceramide glucuronosyltransferase; GMT/GINT, GIPC mannosyl transferase/glucosamine inositolphosphorylceramide transferase; SBH/S4H, sphingoid base hydroxylase/sphinganine-4-hydroxylase.

Plant sphingolipids can be classified in four broad groups: long-chain bases (LCBs), ceramides, glucosyl ceramides (GlcCers), and glycosyl inositol phosphorylceramides (GIPCs) (Zäuner *et al.*, 2010) (Fig. 1). LCBs are the simplest form, and also the defining moiety of all sphingolipids (Warnecke and Heinz, 2003). They are synthesized by condensation of palmitoyl-CoA with serine to generate 3-ketosphinganine, catalysed by a heterodimeric serine palmitoyl transferase (SPT), consisting of LCB1 and LCB2a/b subunits (Tamura *et al.*, 2001; Chen *et al.*, 2006). SPT is regulated by (i) stabilization by a small subunit of serine palmitoyltransferase (ssSPT) (Kimberlin *et al.*, 2013), and (ii) orosomucoids (ORMs), which negatively regulate SPT activity (Chueasiri *et al.*, 2014; Li *et al.*, 2016). Condensation is followed by reduction of the 3-keto-group to a hydroxyl to generate 1,3-dihydrosphingosine (Chao *et al.*, 2011). This LCB, also called sphinganine, can be modified by hydroxylation and/or phosphorylation (Miller *et al.*, 2010). The vast majority of LCBs are converted into ceramides, via an amide linkage to a fatty acid. The fatty acid moiety can be hydroxylated at position C2, and again the C1 position of the LCB moiety can be phosphorylated, here to form a phosphoceramide. Both the LCB and fatty acid moieties of ceramides can be desaturated, thereby producing a wide variety of different structures. Free LCBs and ceramides accumulate at low levels in plant tissues, together presenting <3% of the total sphingolipid pool (Markham and Jaworski, 2007). Both are potent signalling molecules that strongly affect phytohormone levels and serve key roles in programmed cell death (PCD) signalling (reviewed in Berkey *et al.*, 2012).

Ceramides can be further modified by the addition of various polar headgroups, and these more complex sphingolipids can be classified by their linkage to ceramide. Glycosphingolipids contain a glycosidic bond, and in plants are generally present as a single form with a single headgroup, glucose, to make GlcCer (Warnecke and Heinz, 2003). There are exceptions to this; for example, di-glycosylceramides have been detected in the diatom *Thalassiosira pseudonana* (Hunter *et al.*, 2018), spinach leaves (Ohnishi *et al.*, 1983), and pea seeds (Ito *et al.*, 1985). These sometimes contain sugars other than glucose, and so this sphingolipid class is more accurately referred to as hexosylceramides (HexCers).

Phosphosphingolipids have a headgroup linked to ceramide via a phosphodiester bond (Miller *et al.*, 2010). In plants, these are present as a diverse group of membrane lipids, glycosyl inositol phosphorylceramides (GIPCs), which can be extensively modified with a variety of sugars (Mamode Cassim *et al.*, 2019). GlcCers and GIPCs make up ~34% and 64% of the total sphingolipid pool, respectively (Markham *et al.*, 2006), although this ratio varies substantially between species and tissues. Complex sphingolipids are major components of plant cell membranes (Miller *et al.*, 2010), and GIPCs alone may constitute up to 45% of plasma membrane lipids (Cacas *et al.*, 2016). Given their abundance, it is not surprising that GIPCs were recently demonstrated to serve as an important source of phosphate upon phosphate deprivation (Yang *et al.*, 2021).

Both glycosphingolipids and phosphosphingolipids are present across eukaryotes, but take on different forms in different kingdoms. Glycosphingolipids with a single sugar headgroup, such as GlcCer or galactosyl ceramide (GalCer), are present in both plants and fungi (Warnecke and Heinz, 2003). In animals, simple glycosphingolipids with a single sugar are referred to as cerebrosides, and more extensively decorated glycosphingolipids, globosides and gangliosides, are also common (Kolter, 2011). These are enriched in neurons and are known to have essential functions related to cell–cell recognition and signalling (Posse de Chaves and Sipione, 2010). Phosphosphingolipids are arguably even more diverse across kingdoms. GIPCs are distinct and characteristic features of plant cells, though similar structures based on inositol phosphorylceramide (IPC) are also found in fungi (Gronnier *et al.*, 2016). The addition of glucuronic acid (GlcA) to the IPC backbone to generate GlcA-IPC, catalysed by INOSITOLPHOSPHATE CERAMIDE GLUCURONOSYLTRANSFERASE, is specific to plants (Rennie *et al.*, 2014) and these can be, and typically are, further glycosylated. In contrast, IPC, di-IPC, mannosyl-IPC, and mannosyldi-IPC are phosphosphingolipids found in fungi and yeast (Santos *et al.*, 2020). In animals, phosphosphingolipids are present as sphingomyelin bearing a phosphocholine headgroup; these are hallmark components of the myelin sheaths of nerve cell axons.

**Fig. 2.**
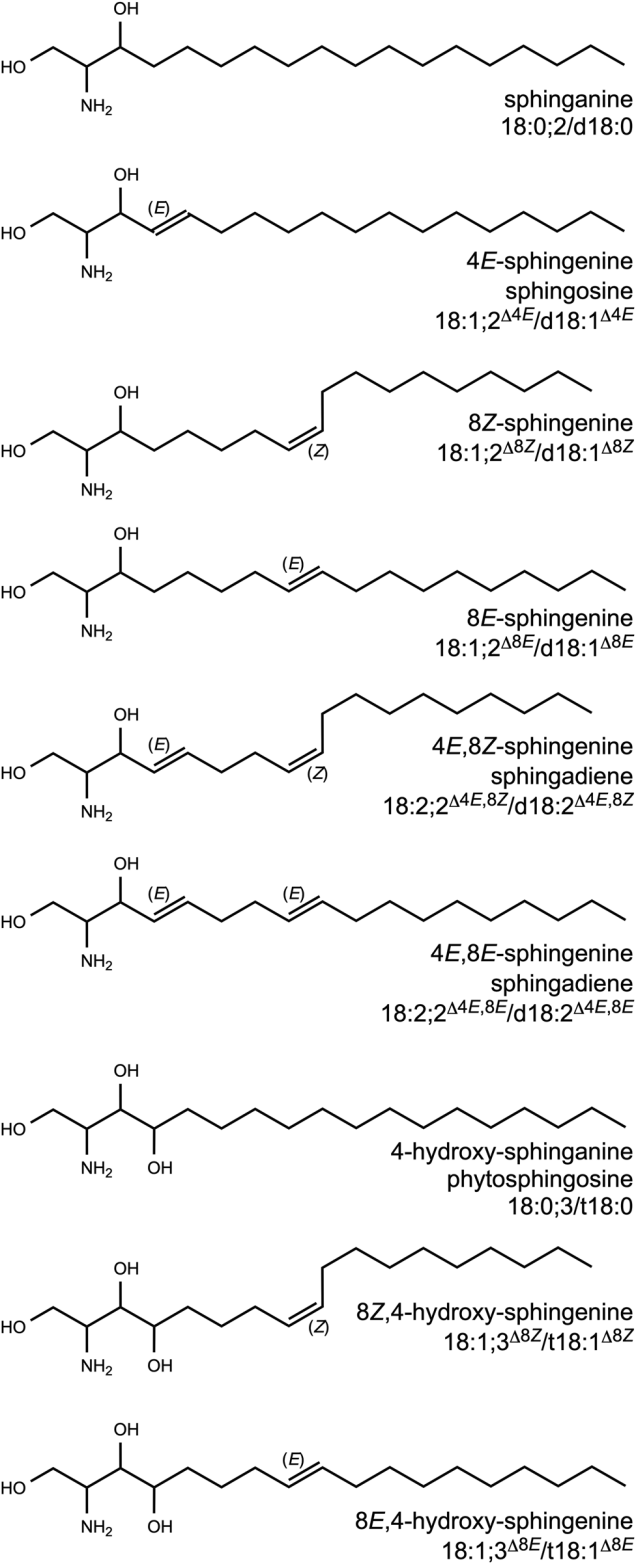
Structures of LCB moieties of plant sphingolipids.

There is also remarkable structural diversity in sphingolipids within the plant lineage alone. This can be expected from a theoretical standpoint, as extensive modifications to the LCB, fatty acid, and headgroup moieties are possible. Empirical data now show that within particular species and even particular tissues, a wide variety of different ceramides are produced and accumulate, both in free form and in complex sphingolipids. The default LCB structure is dihydroxylated, saturated sphinganine (or 1,3-dihydrosphingosine) with the hydroxylations resulting from the original condensation of palmitoyl-CoA and serine (Fig. 2). Trihydroxylated LCB (i.e. t18:0, 4-hydroxysphinganine), produced by SPHINGOID BASE HYDROXYLASE (SBH)/SPHINGANINE-4-HYDROXYLASE (S4H) is commonly referred to as phytosphingosine. t18:0 is common to and abundant in plants; however, its distribution is not strictly limited to the plant lineage. Desaturation at the Δ4 position of the LCB is also possible (sphingosine, or Δ4-sphingenine), but a mutually exclusive alternative to hydroxylation at this site. This Δ4 desaturation, catalysed by DEGENERATIVE SPERMATOCYTE/SPHINGOLIPID Δ4 DESATURASE/DIHYDROCERAMIDE DESATURASE (DES/SΔ4D), is particularly interesting as it is one of few *trans* unsaturations produced in nature (Ternes *et al.*, 2002). Finally, Δ8 unsaturation is also commonly observed in plant LCBs, and can occur in combination with either 4-hydroxylation or Δ4 desaturation. Catalysed by SPHINGOLIPID Δ8 DESATURASE (SLD/SΔ8D), this desaturation is observed in several phyla, including plants, fungi, diatoms, and invertebrates; however, it is especially interesting in plants, where it is unique in that it can produce both *cis*- (*Z*) and *trans*- (*E*) unsaturated products (Sperling *et al.*, 1998; Beckmann *et al.*, 2002; Chen *et al.*, 2012). SLDs in other systems produce only *trans* products (Sato *et al.*, 2019). Modifications of the fatty acid moiety of the ceramide also contribute to sphingolipid structural diversity. These include hydroxylation at the α-position catalysed by FATTY ACYL HYDROXYLASE (FAH) (König *et al.*, 2012; Nagano *et al.*, 2012), desaturation at either the *n*-8 or *n*-9 position catalysed by ACYL-COA DESATURASE (ADS) (Smith *et al.*, 2013) or SPHINGOLIPID FATTY ACYL DESATURASE (SFD) (Resemann *et al.*, 2021), and modification in chain length between 16 and 26 carbon atoms, which is dependent on specificity of the condensing enzyme of the fatty acid elongase complex. Ceramide synthases (CerS/LAG ONE HOMOLOGUEs) link free LCBs to acyl-CoAs to form ceramides, and have specificity for both substrate groups; therefore, they also contribute to ceramide composition in a major way (Markham *et al.*, 2011; Ternes *et al.*, 2011a).

Another source of sphingolipid diversity is the headgroup structure of GIPCs. These vary in the identities and number of glycosylations, with some work indicating that up to 16 sugars can be added to the core GlcA-IPC structure (Cacas *et al.*, 2012). GIPCs are subclassified based on the number of additional sugars added to the GlcA-IPC structure, with A-series bearing one additional sugar, B-series two, etc. The challenges inherent in isolating, detecting, and accurately identifying these structures push the limits of modern analytical methods, although substantial progress has been made in recent years. Profiling of diverse species by Cacas *et al.* (2013) revealed patterns in the number of sugar headgroups added to different lineages. It is equally fascinating that multiple recent studies have uncovered specific functions for specific GIPC modifications in plant physiology and biotic interactions (Grison *et al.*, 2015; Lenarčič *et al.*, 2017; Jiang *et al.*, 2019; Moore *et al.*, 2021).

Molecular genetic studies in selected model organisms have unveiled diversity in sphingolipid modifications among plant lineages, and different physiological and metabolic consequences of sphingolipid modifications. Importantly, novel features uncovered in specific model systems are seldom specific to the lineages in which we first observe or report on them. This highlights the importance of using multiple, diverse model systems in foundational plant research, and cautions against extrapolating generalizations about sphingolipid forms and their metabolic and functional significance. Describing the diversity of plant sphingolipids and determining the physiological relevance of these features is a work in progress.

**Fig. 3.**
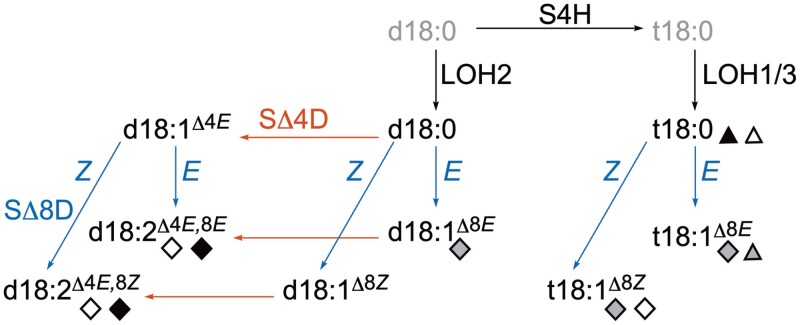
Schematic of LCB modifications occurring on the free base (grey structures) or as a moiety of a ceramide (black structures). SBH/S4H hydroxylates d18:0 to t18:0. Both d18:0 and t18:0 can be substrates for acylation to form ceramides. In red: only d18:0 can be desaturated by SΔ4D, as hydroxylation and desaturation at this position are mutually exclusive. Δ4 unsaturation is only in *E* (*trans*). In blue: d18:1^Δ4*E*^, d18:0, and t18:0 can all be unsaturated by SΔ8D. Products of SΔ8D can be either *Z* (*cis*) or *E* (*trans*). The d18:1^Δ8^ products can also be unsaturated by SΔ4D. LCB structures that are preferentially used in GIPCs are presented with triangles, and in GlcCers with diamonds. Black, *P. patens*; grey, *A. thaliana*; and white, *O. sativa*. Note that although 8*E* and *Z* d18:2 species are indicated as GlcCer species for *P. patens*, the stereochemistry of this double bond remains unknown.

In this review, we will present our current understanding of sphingolipid metabolism based on classical and emerging model systems: the molecular genetic model *Arabidopsis thaliana*, other dicot models that have been extensively chemically profiled including *Solanum lycopersicum* and *Nicotiana tabacum*, the monocot model *Oryza sativa*, and finally the model bryophyte *Physcomitrium patens*. Broad features of the sphingolipid profiles of these models are summarized in Fig. 3. We will discuss selected features of plant sphingolipid synthesis that vary between lineages, with an emphasis on LCB desaturations, fatty acid desaturations, and GIPC headgroups. These findings lay a framework for further exploration of sphingolipid functions in plants.

## Foundational work in *Arabidopsis thaliana*

### Profiling *A. thaliana* sphingolipid content

The variation in physical properties of plant sphingolipids makes it difficult to simultaneously extract and analyse all classes: free LCBs, ceramides, GlcCer, and GIPCs. Additionally, there is inherent difficulty in working with the amphipathic and high molecular weight GIPCs. Profiling of sphingolipids is therefore often carried out on isolated and targeted fractions, for example after TLC separation, or after microsome enrichment in the case of GIPCs. The first global, quantitative analysis of *A. thaliana* sphingolipid content was published by Markham *et al.* (2006). This work systematically compared methods for sphingolipid extraction and thereby provided a basis for future work using rosette leaves from the popular molecular genetic model. Analysis of all sphingolipids was carried out and integrated to allow quantitative comparisons between classes. This work has since been expanded by the analysis of different organs and developmental stages (Luttgeharm *et al.*, 2015; Kehelpannala *et al.*, 2021), and by numerous mutant studies. While the primary focus in Markham *et al.* (2006) was *A. thaliana*, complementary profiling of *Solanum lycopersicum* (tomato) and *Glycine max* (soybean) provided an impression of the chemical diversity present within the dicot lineage (discussed in the following section).

Four methods were compared for optimized sphingolipid extraction; subsequently, neutral and anionic lipids were purified by solid-phase separation, fractionated by preparative HPLC, and finally sphingolipids were analysed by electrospray ionization MS (ESI-MS) (Markham *et al.*, 2006). The LCB moiety profile of neutral sphingolipids (effectively, GlcCers) and anionic sphingolipids (GIPCs) was measured. Neutral sphingolipids include both ceramides and GlcCers, but because GlcCers make up the vast majority of the neutral sphingolipid pool, the neutral lipid profile is primarily representative of these, not of free ceramides. The neutral sphingolipid pool was largely made up of t18:1^Δ8*E*/*Z*^, with a 40% greater amount of Δ8*Z* than *E*, as well as d18:1^Δ8*E*^. Anionic sphingolipids mainly contained t18:1^Δ8*E*/*Z*^, with a 10-fold greater amount of Δ8*E* than *Z*. These differences in the number of hydroxylations, and stereochemistry of the Δ8 double bond, suggest that there are distinct ceramide pools that are shunted into either complex sphingolipid pathway. Because both *cis* and *trans* products are produced by a single type of desaturase in plants, SΔ8D, the identity of the desaturase enzyme itself does not determine the downstream flux into GlcCer or GIPC pools.

### LCB desaturations in *A. thaliana*

Although Δ8 desaturation is a predominant modification in both complex sphingolipid types, blockage of Δ8 desaturation by mutation of the two *SLD/SΔ8D* loci in *A. thaliana* resulted in depletion of GlcCers, and accumulation of GIPCs, with both containing t18:0 in lieu of t18:1^Δ8^. Remarkably, this substantial modification of sphingolipid metabolism resulted in only subtle effects on plant growth under optimal conditions. Mild developmental phenotypes were observed in cold temperatures, suggesting a function for Δ8 desaturation in cold stress tolerance (Chen *et al.*, 2012). Δ4-Desaturated LCBs account for only a small proportion of the total LCBs in *A. thaliana*, and they are only detected in floral tissues. Analysis of loss-of-function *des/sΔ4d* mutants revealed no obvious phenotypes (Michaelson *et al.*, 2009).

Compared with complex sphingolipids, the free ceramide pool of leaves has unique LCB content, made up of roughly equimolar amounts of t18:1^Δ8*E*^ and t18:0 (Markham *et al.*, 2006). This may suggest that desaturation of the LCB moiety of ceramides could be a determinant or a limiting factor for complex sphingolipid synthesis. Alternatively, this could be interpreted as evidence that ceramide pool composition is only partially determined by flux into downstream products.

### Fatty acyl content of sphingolipids in *A. thaliana*

Profiles of the fatty acid moieties of free ceramides, GlcCers, and GIPCs are also unique, reflecting differences in the activity of ceramide synthases, fatty acid desaturases, and fatty acid hydroxylases. Broadly, in *A. thaliana*, there is an enrichment of C_22–26_ very-long-chain fatty acids (VLCFAs) in GIPCs, and in GlcCers there is a split between C_16_ long-chain fatty acids and C_24_ VLCFAs (Markham *et al.*, 2011). This trend is strongly influenced by the specificity of the ceramide synthases LOH1/2/3 [named after longevity assurance gene (LAG) One Homologue] for both their LCB and fatty acyl-CoA substrates, with LOH1 and LOH3 showing preference for trihydroxy-LCB and VLCFA substrates, and LOH2 for dihydroxy-LCB and LCFA substrates (Markham *et al.*, 2011; Ternes *et al.*, 2011a).

The fatty acid moieties of most free ceramides, GlcCers, and GIPCs are hydroxylated at the α-position in *A. thaliana*; the enzymes responsible for this modification, FATTY ACID HYDROXYLASE (FAH) 1/2, have been characterized by mutant analysis (Mitchell and Martin, 1995; König *et al.*, 2012; Nagano *et al.*, 2012). Double mutants had reduced GlcCer content and accumulated LCBs and free ceramides, suggesting that ceramide hydroxylation influences complex sphingolipid assembly. GIPCs were not measured in this analysis. The *fah1* single mutant was hypersensitive to oxidative stress, and the double mutant had a mild growth defect and enhanced resistance to a biotrophic pathogen, together suggesting a function for sphingolipids harbouring α-hydroxylated VLCFAs in stress responses (König *et al.*, 2012; Nagano *et al.*, 2012).

The VLCFA moieties of *A. thaliana* sphingolipids are also often monounsaturated, in free ceramide, GlcCer, and GIPC pools. The primary desaturase required for this modification is ACYL-COA DESATURASE-LIKE2 (ADS2) (Smith *et al.*, 2013), based on the characterization of mutant and overexpression lines. The desaturase is presumed to use acyl-CoA substrates, as the strongest effects of its genetic manipulation were observed in the acyl-CoA pool, and influenced both glycerolipid and sphingolipid content. The *ADS* gene family consists of nine members in *A. thaliana*, most of which have been only partially characterized by heterologous expression in yeast.

### Regulation of free LCB and ceramide content in *A. thaliana*

While the vast majority of sphingolipids synthesized in *A. thaliana* are destined to become complex sphingolipids via the addition of sugar headgroups, a small fraction remains as free LCBs and ceramides. These simpler forms are metabolic fates with their own physiological functions. LCBs and ceramides are potent signalling molecules, with activities dependent on their phosphorylation status (reviewed in Berkey *et al.*, 2012; Luttgeharm *et al.*, 2016). LCBs and LCB phosphates (LCB-Ps) are broadly considered to have antagonistic activity in cell death signalling, with LCBs triggering cell death, and LCB-Ps mitigating or down-regulating this process (Berkey *et al.*, 2012). However, this antagonism was recently called into question by Glenz and co-workers, who demonstrated that competitive uptake of the phosphorylated and non-phosphorylated forms could account for some of the observed antagonism of LCB-Ps against LCB-induced PCD (Glenz *et al.*, 2019). This important physiological response certainly requires further investigation, particularly the identification of additional genetic components associated with PCD signalling. The kinases responsible for phosphorylating LCBs, SPHINGOSINE KINASE 1 and SPHINGOSINE KINASE 2, have primarily been studied in *A. thaliana* (Coursol *et al.*, 2005; Worrall *et al.*, 2008; Alden *et al.*, 2011; Guo *et al.*, 2011). LCB-Ps can be depleted either by dephosphorylation by LONG-CHAIN BASE PHOSPHATE PHOSPHATASEs (LCB-PP1 and LCB-PP2) (Worrall *et al.*, 2008) or by cleavage of the LCB backbone to produce hexadecanal and phosphoethanolamine by LONG-CHAIN BASE PHOSPHATE LYASE (DPL) (Tsegaye *et al.*, 2007).

Phosphorylation of ceramide is catalysed by a ceramide kinase, ACCELERATED CELL DEATH5 (ACD5) (Greenberg *et al.*, 2000; Liang *et al.*, 2003; Wang *et al.*, 2008). A phosphatase catalysing the dephosphorylation of ceramide phosphates has not been reported in plants.

There are several families of ceramidases that can hydrolyse ceramides to their LCB and fatty acid components; these are classified as acid, neutral, or alkaline ceramidases based on the optimal pH for their activity. To date, only neutral and alkaline ceramidases have been identified and characterized in plants. Their activities are tightly associated with both pathogen-triggered PCD-like signalling as well as developmentally controlled PCD, autophagy, and cellular turgor pressure (Chen *et al.*, 2015; Li *et al.*, 2015; Wu *et al.*, 2015; Zheng *et al.*, 2018; Zienkiewicz *et al.*, 2020). Interestingly, some ceramidases also have the capacity to synthesize ceramides by reverse activity (Okino *et al.*, 2003).

### Complex sphingolipids in *A. thaliana*

Identification of *GCS* in *A. thaliana* was enabled by homology to *GCS* genes in fungi and animals. However, characterization of a *gcs* mutant and delineation of the physiological role of GlcCers was impeded by its severe, seedling-lethal phenotype (Msanne *et al.*, 2015). In contrast to the phenotype of mutant seedlings, mutant calli grew normally, suggesting that *gcs* mutants are impaired in cellular differentiation. Despite this deleterious effect of full loss of function on plant viability, GlcCer content reduced to as low as 2% of wild-type levels by RNAi was sufficient to maintain viable, fertile plants, albeit with stunted development (Msanne *et al.*, 2015). Another striking feature of *gcs* mutants was that they did not strongly accumulate ceramide substrates of GCS, instead incorporating ceramide species typically associated with GlcCers into GIPCs. This suggests that ceramide accumulation was not the cause of their severe developmental phenotype. While GlcCers make up approximately one-third of the sphingolipid content of *A. thaliana* leaves, their content is enriched 8-fold in pollen. The reason for this stark difference between sporophyte and male gametophyte generations remains unclear and requires further investigation (Luttgeharm *et al.*, 2015).

The initial characterization of GIPC structures was established from corn and tobacco in the mid 1900s (Carter *et al.*, 1958; Kaul and Lester, 1975); more recent work with ESI-MS/MS and MALDI-MS has revealed a wide diversity of GIPC structures within and among different plant species (Cacas *et al.*, 2013; Buré *et al.*, 2014). Product and precursor ion scans of the major anionic sphingolipids of *A. thaliana* by Markham *et al.* (2006) suggested a headgroup structure of hexose–hexuronic acid–inositol phosphoceramide—an A-series GIPC. Precise identification of the headgroups was not possible with the ESI-MS approach used here. Subsequent work combining ESI-MS/MS with MALDI-MS/MS resolved the headgroup profiles of GIPC structures in detail, for both *A. thaliana* leaves and cell cultures (Buré *et al*., 2011, 2016).

IPCs are common to plants, fungi, and protists (Gronnier *et al.*, 2016). The genes responsible for transfer of the inositol phosphate headgroup to ceramide was identified previously in *Saccharomyces cerevisiae* (*AUR1*) (Nagiec *et al.*, 1997) and in several protozoans (*INOSITOL PHOSPHATE CERAMIDE SYNTHASE*, *IPCS*) (Denny *et al.*, 2006); however, the first plant *IPCS* was identified serendipitously via a forward genetic screen for enhanced disease responses (Wang *et al.*, 2008). Three paralogues in *A. thaliana* have since been characterized (Mina *et al.*, 2010). Total loss of function is impossible to assess, as triple knockouts are lethal, and single knockouts do not show any obvious defects. Curiously, several additional proteins with sequence similarity to IPCSs, named PHLOEM LOADING MODULATORs (PLMs), have also been identified in *A. thaliana*, which are required for plasmodesmal function (Yan *et al.*, 2019). The biochemical function of PLMs remains unknown, and the specific physiological functions and biochemical specificities of IPCSs have yet to be distinguished.

In contrast to *IPCS* genes, the gene responsible for transferring the hexuronic acid moiety, determined to be glucuronic acid (GlcA) (Kaul and Lester, 1975), *IPUT*, was identified by reverse genetics (Rennie *et al.*, 2014). Its product is encoded by a single gene in *A. thaliana*, and is thought to be plant specific (Cacas *et al.*, 2012). A null *iput* mutant could not be obtained, as the mutant lesion was found to be non-transmissible through pollen due to a defect in ovule targeting (Tartaglio *et al.*, 2017). Transient complementation of the mutation with a pollen-specific promoter enabled characterization of mutant sporophytic tissue, which had developmental defects (Tartaglio *et al.*, 2017). Recently, the partial loss-of-function *moca1* allele of *IPUT1* was identified in a forward genetic screen for ionic stress sensitivity, implicating GIPCs in salt sensing and initiation of cellular responses to ionic stress (Jiang *et al.*, 2019).

The identity of the hexose residues added onto GlcA of the GIPC was explored by Mortimer *et al.* (2013), during the isolation and characterization of a Golgi nucleotide-sugar transporter (GONST) that yielded many surprising and interesting results. Similar to other sphingolipid-deficient mutants, *gonst1* mutants have a dwarf phenotype and elevated salicylic acid levels, resulting in spontaneous lesion formation. Recent work on a close homologue of *GONST1*, *GONST2*, revealed similar activities for the two genes *in planta*, but different functions determined by their expression profiles. This work also highlighted the effect of disruptions to GIPC synthesis on plant susceptibility to biotrophic pathogens, here *Golovinomyces orontii* (Jing *et al.*, 2021). Biochemical characterization of *gonst1* revealed that the loss of this transporter, which has the capacity to transport GDP-mannose, -galactose, and -fucose sugars, resulted in an ~75% reduction in Hex-GIPC, and an exponential increase in GlcA-IPC lacking further glycosylation. Interestingly, monosaccharide composition analysis of isolated Hex-GIPC headgroups identified arabinose, galactose, glucose, and mannose, but only mannose content was reduced in the *gonst1* mutant. In line with this, characterization of GIPC MANNOSYL TRANSFERASE (GMT) mutants, defective in the enzyme required for the transfer of the hexose onto GIPC, revealed the strongest reduction in mannose content in the mutants. Again, other sugar moieties were detected in GIPCs, with glucose particularly abundant and variable in content (Fang *et al.*, 2016). In both of these studies, it was concluded that mannose-GlcA-IPC is the major Hex-GlcA-IPC (and GIPC, overall) species present in *A. thaliana*. It remains unclear how depletion of the supply of mannose for addition to GIPCs in both of these mutants results in such a strong decrease in total Hex-GIPCs, when other sugars were also detected as abundant hexose moieties in the headgroup.

## Expanded insight from other dicots

### Profiles of non-models and insight into functions of LCB desaturation

Profiling of the sphingolipid content of *S. lycopersicum* and *G. max* by Markham *et al.* (2006) revealed substantial variation in sphingolipid content within the dicot lineage. Within the neutral sphingolipids, an entirely different LCB moiety predominated in *S. lycopersicum*, d18:2^Δ4*E*,Δ8*E/Z*^. In *G. max*, both the LCB most abundant in *A. thaliana*, t18:1^Δ8*E*/*Z*^, and that most abundant in *S. lycopersicum*, d18:2^Δ4*E*,Δ8*E/Z*^, were observed. The significance of these differences in LCB moieties remains unclear. In contrast to the variation in ceramide structure of GlcCers, the predominant ceramide backbone of GIPCs in all three species studied by Markham *et al.* (2006) was t18:1/h24:0; from this, it can be suggested that trihydroxylated LCBs have conserved use in GIPC assembly.

The absence of d18:1^Δ4^ (sphingosine) as a major LCB moiety in any of these model plants stands in stark contrast to animals, where sphingosine is an important and abundant LCB. In animals, its derivative sphingosine-1-phosphate (S-1-P) is known to be a potent signalling molecule (Hannun and Obeid, 2018). Low levels of S-1-P have been detected in plants, and these increase upon drought stress (Ng *et al.*, 2001). Application of S-1-P to *Commelina communis* (Asiatic dayflower) and *A. thaliana* has been reported to affect stomatal guard cell turgor in response to drought stress (Ng *et al.*, 2001; Coursol *et al.*, 2003). Whether it is a physiologically relevant signalling molecule remains uncertain since, as mentioned, *A. thaliana* Δ4 desaturase (*des/sΔ4d*) mutants unable to produce sphingosine and S-1-P did not have any obvious growth defects (Michaelson *et al.*, 2009).

The distribution of LCB moieties that predominate in different plant lineages was explored in detail by Islam *et al.* (2012). Here, the desaturation of di-hydroxy LCBs was surveyed across a broad sampling of species. Unsaturation of Δ4 was the primary monounsaturation in sampled non-seed plants and angiosperms, while Δ8 unsaturation was detected as the predominant monounsaturated LCB only in some select angiosperm species, including *A. thaliana*, and was predominant in gymnosperms. The authors concluded that Δ4 monounsaturation is ancestral. Very recently, Zhang (2021) proposed that the availability of dihydroxy LCB substrate is a major factor determining whether the Δ4-unsaturated LCB moiety accumulates, and demonstrated in *A. thaliana* that modifying the amount of ceramides bearing dihydroxy LCB moieties resulted in changes in the activity of a heterologously expressed Δ4 desaturase. However, due to the high activity level of the endogenous Δ8 desaturase, the Δ4 product accumulating in these transgenic lines was d18:2. Regardless of the mechanistic reason for Δ4- or Δ8-unsaturated LCB accumulating, the physiological function of Δ4-unsaturated LCB remains of interest. Islam *et al.* (2012) suggested that investigation of a model plant accumulating Δ4-monounsaturated LCBs, in addition to Δ8-accumulating *A. thaliana*, would be useful to improve our understanding of plant sphingolipid metabolism in general. To date, the only other species in which Δ4 desaturase mutants have been characterized are *O. sativa* and *P. patens* (Sato *et al.*, 2019; Gömann *et al.*, 2021a), discussed in the following sections.

### Use of inhibitors of sphingolipid metabolism and insight into reaction order

Profiling sphingolipid content in diverse model and non-model species can be especially insightful when paired with the application of inhibitors of sphingolipid assembly. Inhibitors are valuable tools in these systems that are less tractable by genetic manipulation, which nevertheless offer specific advantages with respect to their development and physiology. Inhibitors often cause growth and developmental defects that mimic mutants defective in sphingolipid assembly. Many chemicals have been identified that affect sphingolipid metabolism, including several mycotoxins that inhibit ceramide synthesis (Chen *et al.*, 2020), 1-phenyl-2-decanoylamino-3-morpholino-1-propanol (PDMP), which inhibits GlcCer synthesis (Msanne *et al.*, 2015), and myriocin, which inhibits LCB synthesis (Miyake *et al.*, 1995; Chen *et al.*, 2021), among others. The ceramide synthase inhibitors fumonisin B_1_ (FB_1_) from *Fusarium*, and *Alternaria alternata lycopersici* (AAL) toxin, have been particularly well used in plant research. Treatment of duckweed (*Lemna pausicostata*), *S. lycopersicum* plants, *S. lycopersicum* leaf discs, or *N. tabacum* callus with either FB_1_ or AAL toxin consistently resulted in the accumulation of d18:0 and t18:0 LCBs (Abbas *et al.*, 1994). Other sphingoid bases could be detected in small amounts after these treatments, but their deduced structures were inconsistent with known LCBs. Direct treatment of *L. pausicostata* cultures with d18:0, t18:0, and d18:1^Δ4^ LCBs produced similar growth defects to the mycotoxins, supporting the notion that FB_1_ and AAL truly act as ceramide synthase inhibitors, and that their toxic effects are mediated by excessive accumulation of LCB substrate upon ceramide synthase inhibition (Tanaka *et al.*, 1993).

The effect of these mycotoxins on sphingolipid content may also help to reveal the order of reactions during ceramide assembly; accumulation of only d18:0 and t18:0 upon FB_1_ or AAL treatment suggests that hydroxylation occurs on LCB substrates, and that desaturation, at either the Δ4 or Δ8 position, uses ceramide substrates. This model is now also supported by mutant studies in *A. thaliana.* In the hydroxylase mutant *sbh1 sbh2*, there is exponential accumulation of di-hydroxylated free LCBs and all sphingolipids incorporating di-hydroxylated LCB moieties, as well as a total depletion of tri-hydroxylated LCBs and sphingolipids containing tri-hydroxylated LCB moieties (Chen *et al.*, 2008). In contrast, in the *sld1 sld2* mutant lacking the Δ8 desaturase (SLD/SΔ8D), the ceramides, GlcCers, and GIPCs with t18:0 LCB moieties accumulate exponentially, at the expense of t18:1, while the free saturated LCBs show only a modest increase (Chen *et al.*, 2012). Cumulatively, these data present a logical and consistent model of ceramide assembly and modification.

### Diversity in headgroup composition

Beyond the variations in the ceramide content of different plant species, vast chemical diversity is observed in the headgroup content of the GIPCs. Structural diversity in GIPC headgroups has arguably been best described in *N. tabacum* (Buré *et al.*, 2011; Cacas *et al.*, 2016) and derived Bright Yellow 2 (BY-2) culture lines, owing to the utility and establishment of BY-2 in other fields of research, and the simplicity of this cell line as a source of uniform, non-differentiated plasma membrane. In contrast to the Hex-GlcA-IPC content of *A. thaliana*, the main species of GIPCs in *N. tabacum* were determined to be hexamine-GlcA-IPC and *N*-acetylated hexamine-GlcA-IPC (Buré *et al.*, 2011). These studies of cell cultures have consistently revealed an increase in the extent of glycosylation when compared with developed organs containing multiple cell and tissue types; enrichment of detergent-insoluble membranes from BY-2 cells revealed that GIPCs could constitute up to 60% of the lipids in this fraction. The roles of GIPCs in the functionality of these membrane domains is poorly understood, but an exciting and growing field (Gronnier *et al.*, 2016; Mamode Cassim *et al*., 2019, 2021).

## Clues from a model monocot: *Oryza sativa*

### Profiling *O. sativa* sphingolipid content and insight into LCB desaturations

Ishikawa *et al.* (2016) replicated the methodology from Markham *et al.* (2006) for analysis of the *O. sativa* sphingolipidome. An additional step in their processing was the enrichment of sphingolipid classes with commercial solid-phase extraction (SPE) cartridges. Similar to *G. max*, *O. sativa* accumulates neutral sphingolipids with both d18:2^Δ4*E*, Δ8*E/Z*^ and t18:1^Δ8*E*/*Z*^. The cumulative data suggest that there is substantial flexibility in the identity of the ceramide backbone used for GlcCer synthesis, and that this does not show any obvious lineage-specific correlations. Although d18:1 is a minor component of GlcCers and free ceramides, the predominant desaturation in both classes was at the Δ4 position, conforming to the trends reported by Islam *et al.* (2012). The GIPC content of *O. sativa* was distinct from that of the dicots described above in that its predominant backbone structure was t18:0, not t18:1. The t18:0 backbone made up >80% of the GIPC class. *O. sativa* GlcCer Δ8 unsaturation was primarily *Z*, and *E* in the GIPCs. Building on this information in their characterization of *O. sativa* SLD/SΔ8D and DES/SΔ4D enzymes, Sato *et al.* (2019) suggested that generally, *Z* (*cis*) unsaturated LCBs are utilized for GlcCer synthesis, while saturated and *E* (*trans*) unsaturated LCBs are used for the synthesis of GIPCs.

In terms of the overall accumulation of *Z* or *E* Δ8 unsaturation, there is a clear comparison that can be made between *A. thaliana* and *O. sativa*: in *O. sativa* there is a greater overall accumulation of Δ8-unsaturated LCB moieties in *Z*, while *A. thaliana* accumulates more *E*. This trend is conserved when the *O. sativa* and *A. thaliana* SLD/SΔ8D are expressed in yeast, suggesting that the *E*/*Z* ratio of LCB moieties in a given species is directly related to the SLD/SΔ8D enzyme activity, not downstream utility or other factors (Sato *et al.*, 2019). Because *Z* unsaturation lends greater flexibility/fluidity to lipid membranes than *E* (*trans*), Imai *et al.* (1997) hypothesized that the tendency of a given plant’s SLD/SΔ8D(s) to produce either isomer could be related to its cold hardiness. They performed a survey of 22 vascular plant species, sampling both chilling-tolerant and chilling-sensitive species. Although they confirmed their expectation that chilling-tolerant plants accumulated more *Z* than *E*, there was no correlation between the chilling sensitivity of the sampled plants and their LCB isomer profile. Notably, mutation of the *SLD/SΔ8D* in *O. sativa* is lethal (Sato *et al.*, 2019), in contrast to the weak phenotype of *A. thaliana SLD/SΔ8D* mutants (Chen *et al.*, 2012). Perhaps the added membrane fluidity provided by the *Z* Δ8 double bond in *O. sativa* is required for viability, while in *A. thaliana* other membrane components are sufficient to maintain membrane integrity and function.

Overexpression or ectopic expression of *O. sativa* and *A. thaliana* SLD/SΔ8D in *O. sativa*, respectively, yielded surprisingly different results. *Oryza sativa* SLD/SΔ8D (*Z*-producing) overexpression had only minor effects on the overall sphingolipid profile of transgenic plants, while ectopic expression of *A. thaliana* SLD/SΔ8D (*E*-producing) produced exponentially more t18:1-GIPC, at the expense of t18:0-GIPC. This suggests that the *E*/*Z* ratio of products of SLD/SΔ8D could have a strong influence on the use of either t18:0 or t18:1 for GIPCs. That is, *trans* t18:1 or saturated t18:0 may be more acceptable to IPCS for GIPC synthesis than *cis* t18:1; by this logic, species with desaturases producing more *cis* product would use t18:0 in GIPCs, and species with desaturases producing more *trans* product would utilize this t18:1 in GIPCs. As *cis* unsaturation introduces more fluidity to membrane bilayers, this generalization supports a model wherein GIPCs are major components of detergent-insoluble membranes (Sato *et al.*, 2019).

### Fatty acyl unsaturation in sphingolipids of *O. sativa*

A particularly striking feature of the *O. sativa* sphingolipid profile in comparison with *A. thaliana* is the complete absence of unsaturated fatty acid moieties in all sphingolipid classes: free ceramides, GlcCers, and GIPCs. Fatty acyl unsaturation is a common and often prevalent feature of some profiled plant species, where it has been suggested (Fukuchi-Mizutani *et al.*, 1998; Smith *et al.*, 2013) to have a physiological role in cold stress adaptation. *Oryza sativa* is not unique in lacking ceramide fatty acyl unsaturation; unsaturated fatty acyl moieties are also absent from both GlcCers and GIPCs of tobacco (Cacas *et al.*, 2016), and GlcCers of lupin bean and mung bean lack unsaturated fatty acids (Adem *et al.*, 2021). Comparison of GlcCer profiles (Lynch and Dunn, 2004) from *Spinacia oleracea* (spinach) leaf (Ohnishi *et al.*, 1983), *Zea mays* (maize) leaf (Ohnishi *et al.*, 1988), *Secale cereale* (rye) leaf (Cahoon and Lynch, 1991), *Triticum aestivum* grain (Fujino and Ohnishi, 1983), and *G. max* (Ohnishi and Fujino, 1982) revealed accumulation of unsaturated fatty acids (hydroxy-24:1) only in *S. cereale*, which the review authors associated with this species’ cold hardiness. This correlation warrants further exploration through metabolic profiling: in more species, under cold stress conditions, and in the GIPCs of the species previously only profiled for GlcCers. Importantly, in their characterization of *A. thaliana ADS2*, Smith *et al.* (2013) did note that the *ADS* gene family appeared to be lacking in monocots, with no homologues found in the genomes of *O. sativa*, *Brachypodium distachyon*, or *Z. mays*; thorough analysis of the phylogeny of the *ADS* gene family will therefore also be essential to understanding the role of sphingolipid fatty acyl unsaturation in plant biology.

### GIPC headgroup assembly in *O. sativ*a

Analysis of the GIPC content of *O. sativa* (Ishikawa *et al.*, 2016) revealed an abundance of GIPCs with two additional sugar residues added on to the glucuronic acid—B-series GIPCs—compared with the predominance of A-series GIPCs described in *A. thaliana* (Mortimer *et al.*, 2013) and *Nicotiana benthamiana* (Cacas *et al.*, 2016). The accumulation of B-series GIPCs is a general trend observed in monocots, whereas dicots typically accumulate more A-series (Cacas *et al.*, 2013). This is now recognized to have physiological significance in the context of plant–pathogen interactions. Necrosis- and ethylene-inducing peptide-1-like (NLP) proteins, which are produced by many bacterial, fungal, and oomycete pathogens and are necessary for infection, were found to directly bind GIPC headgroups. The distinction between A- and B-series has been demonstrated to be a determining factor for NLP binding, and therefore for pathogenesis (Lenarčič *et al.*, 2017).

Another interesting feature of the GIPC profile of *O. sativa* (Ishikawa *et al.*, 2016) is the abundance of hexosamine (HexN)/*N*-acetylated hexosamine (HexNAc)-containing headgroups, which prompted the investigation of the glycosyltransferases responsible for adding these moieties (Ishikawa *et al.*, 2018). As described in the previous section, HexN/HexNAc as moieties of GIPCs have also been detected in dicots, for example *N. tabacum* (Buré *et al.*, 2011), and also specifically in seeds and flowers of *A. thaliana* (Tellier *et al.*, 2014; Luttgeharm *et al.*, 2015); the preference for Hex versus HexN/HexNAc is therefore not specific to the dicot or monocot lineages. However, the addition of these two headgroup moieties has been thoroughly compared and investigated in *O. sativa*, and so we will address this topic here. In contrast to the diversity of possible Hex substrates for Hex-GIPC synthesis, glucosamine (GlcN)/*N*-acetylated glucosamine (GlcNAc) is the only hexosamine detected in plants, and it is therefore assumed to be the moiety present on GIPCs (Ishikawa *et al.*, 2018). UDP-GlcNAc is abundant in plants, while UDP-GlcN has not been detected (Ito *et al.*, 2014); therefore, Ishikawa *et al.* (2018) predicted that GlcNAc is transferred onto GlcA-IPC, and that GlcN-GlcA-IPC is a product of deacetylation on the assembled GIPC.

The glycosyltransferases responsible for transfer of Hex, GMT, and HexNAc, GINT, belong to the same carbohydrate-active enzyme (CAZy) glycosyltransferase family, GT64. However, there is a great deal of variety within this family. The enzymes responsible for these transfers in *A. thaliana* are only 29% identical; AtGINT is approximately double the length of AtGMT, and contains an additional exostosin domain. The phenotypes of *A. thaliana gmt* (Fang *et al.*, 2016) and *O. sativa gint* (Ishikawa *et al.*, 2018) mutants were similar, with stunted growth and development, and cell death lesions. However, there are multiple lines of evidence that the distinct presence or profile of either product Hex-GlcA-IPC or HexNAc-GlcA-IPC is physiologically relevant. Complementation of *A. thaliana gmt* mutants with either *A. thaliana* or *O. sativa GINT* recovered total GIPC levels, with GlcNAc replacing Hex, but only partially rescuing the developmental phenotype of these mutants. Further evidence of the significance of Hex/HexNAc content of GIPCs was recently found in the roots and nodules of the model legume *Medicago truncatula* by Moore *et al.* (2021). *MtGINT1*, the gene required for transferring HexNAc headgroups to GIPCs, is expressed in tissues synthesizing perimicrobial membranes that directly interact with both bacterial (nodulating rhizobia) and fungal (arbuscular mycorrhiza) root symbionts. *MtGINT1*, and presumably its HexNAc-GIPC products, was found to be required for maintaining these important symbioses.

## And a model moss: *Physcomitrium patens*

### Profiling *P. patens* sphingolipid content and analysis of mutant phenotypes

The sphingolipid profile of the model bryophyte *Physcomitrium patens* was recently published, lending greater diversity to the list of examined plant species (Resemann *et al.*, 2021). The methods used were based on Markham *et al.* (2006) and Tarazona *et al.* (2015). Major ceramide species are t18:0 LCB linked to hydroxy-22:0, -24:0, and -24:1 fatty acids. The GlcCer content of *P. patens* consisted almost entirely of d18:2 LCB with hydroxy-20:0 fatty acid. This is a unique feature of the *P. patens* sphingolipidome in comparison with other investigated plants, from the perspective that the LCB and fatty acid moiety of GlcCer is so homogeneous. The GIPCs used t18:0 exclusively as an LCB moiety, amide-linked to hydroxylated 20:0, 22:0, 24:0, and 24:1 fatty acids. Both A- and B-series GIPCs were detected, with both Hex and HexNAc headgroups. Mutant analysis by Steinberger *et al.* (2021) reported a greater variety of GIPC headgroups, including one C-series GIPC with Pent-Hex-Hex. C- and D-series GIPCs containing a mixture of pentose and hexose sugars in *P. patens* were also reported by Cacas *et al.* (2013). The discrepancy among these reports of the GIPC content of *P. patens* highlights the difficulty inherent in targeted profiling of an essentially unknown target; although tremendous progress has been made in this field, establishment of robust and reproducible extraction and measurement is very much a work in progress.

Standard methods for cultivating *P. patens* gametophytic tissues *in vitro*, with the possibility of manipulating development by nutritional supplementation, or complementing metabolic deficiencies, have proven to be valuable tools for the analysis of sphingolipid-deficient mutants that have severe developmental defects. Two groups recently and independently generated and characterized *P. patens* mutants deficient in the LCB hydroxylase SPHINGANINE C-4 HYDROXYLASE (S4H)/SPHINGOID BASE HYDROXYLASE (SBH) (Gömann *et al.*, 2021b; Steinberger *et al.*, 2021). The analogous *sbh1 sbh2* loss-of-function mutant of *A. thaliana* was unable to develop beyond early vegetative growth, necessitating the use of RNAi to characterize the effects of a deficiency in tri-hydroxylated LCBs. In contrast, the *P. patens* mutants were viable and could be cultivated in parallel with wild-type plants. The simple developmental patterning of *P. patens* also facilitated the analysis of mutant phenotypes affecting growth, cell division, and differentiation. As severe, non-viable, and/or pleiotropic phenotypes, as well as complex developmental patterning, have presented major obstacles to the analysis and characterization of many sphingolipid-deficient vascular plant mutants, the simplicity of *P. patens* makes it an appealing model for further work on sphingolipids, both as structural components of cell membranes and as essential signalling molecules.

### GlcCer assembly in *P. patens*

The homogeneous GlcCer content of *P. patens* facilitated further investigation of the order and importance of reactions required for GlcCer synthesis in this species, via characterization of loss-of-function *glucosyl ceramide synthase* (*gcs*) and *sphingolipid delta 4 desaturase* (*des/sΔ4d*) mutants (Gömann *et al.*, 2021a). Multiple alleles of both single mutants were nearly completely devoid of GlcCers, and *sΔ4d* mutants additionally lacked any LCB unsaturation, at either Δ4 or Δ8. This result suggests that in *P. patens*, Δ4 unsaturation precedes Δ8 desaturation, and is a prerequisite for both Δ8 desaturation and addition of the hexose headgroup. A similar result was obtained and similar conclusions were drawn from study of *Pichia pastoris* (Ternes *et al.*, 2011b). In contrast, in *A. thaliana*, which largely accumulates Δ8-monounsaturated LCBs in lieu of Δ4 as moieties of free ceramides, GlcCers, and GIPCs, Δ8 unsaturation is not required for Δ4 desaturase activity (Chen *et al.*, 2012). Perhaps a better comparison for *PpsΔ4d* is with *Osdes* mutants, as *O. sativa* also accumulates Δ4-unsaturated LCB moieties. In *Osdes*, Δ8-unsaturated LCBs accumulate to compensate for the loss of Δ4,Δ8-di-unsaturated LCBs, indicating that Δ4 unsaturation is not a universal prerequisite for Δ8 unsaturation in species where Δ4 unsaturation is prevalent. However, this is only true in total LCB analysis. The loss of Δ4 unsaturation sharply reduces the production of GlcCers, which primarily contain d18:2 LCBs (Sato *et al.*, 2019). Surprisingly, a similar observation was reported in the analysis of the *A. thaliana sΔ4d* mutant. In this mutant, in a species where Δ4 appears to have less physiological consequence, there was a substantial reduction in the total amount of GlcCer, which exceeded the specific reduction in d18:2 (Michaelson *et al.*, 2009). Altogether, these results present a mixed model of the importance of Δ4 versus Δ8 LCB desaturation relative to each other, but suggest that Δ4 desaturation is broadly preferred for GlcCer assembly. It remains unclear whether these observations are due to substrate specificity of the downstream enzymes, metabolite channeling into different pools with different metabolic fates, or interaction and/or regulation between the enzymes.

Strikingly, although both *Ppgcs* and *PpsΔ4d* mutants were nearly completely devoid of GlcCers, with *sΔ4d* accumulating only trace amounts of GlcCer, the *gcs* mutants presented severe developmental defects while *sd4d* mutants were effectively indistinguishable from the wild type. The authors speculated that this contrast could be due to either accumulation of ceramides in the *gcs* mutant, or the trace quantities of GlcCer in *sd4d* being sufficient to maintain plant health. Generation and characterization of a double mutant will be essential to answer this question (Gömann, 2020). Results from *A. thaliana gcs*, which did not accumulate ceramide precursors but nevertheless presented a strong developmental phenotype, would hint that the latter hypothesis is more likely.

### Sphingolipid fatty acyl unsaturation in *P. patens*

Unsaturated fatty acids were abundant moieties of ceramides and GIPCs, and were detected in trace amounts in non-d18:2/h20:0 GlcCer. The detection of unsaturated fatty acids in *P. patens* could be argued to fit with the requirement for this modification for cold hardiness. This notion was further explored via characterization of the *P. patens* SPHINGOLIPID FATTY ACYL DESATURASE (SFD; Resemann *et al.*, 2021). Remarkably, this desaturase is from an entirely distinct desaturase family from the characterized *A. thaliana* ADS2 catalysing the analogous reaction. Sphingolipid fatty acid desaturation, in both of these clearly distinct model systems, catalysed by clearly distinct enzymes, may be similarly required for adaptation to cold stress. This points to convergent evolution of mechanisms of cold stress tolerance, between bryophyte and vascular plant lineages which diverged >500 million years ago. Further investigation of both the *ADS* and *SFD* families is warranted, as little is known about both and as yet based on very few representative members. Additionally, the distribution of these enzymes, and whether these or another as yet unidentified group of desaturase enzymes is present in monocots will be essential for our understanding of the function of sphingolipid fatty acyl desaturation in plants.

## Outlook

Our understanding of plant sphingolipid metabolism has expanded in recent years, as a product of substantial, focused work by many research groups. While chemical profiling of sphingolipids in various plants has been done for some time, more recently the increased accessibility of non-model systems for genetic manipulation has provided a convenient, complementary toolkit. Pairing exploration via these two approaches will provide greater insight into both sphingolipid metabolism and function in plants. Further, tools that are flexible in the choice of a host will enable targeted investigations where unexpected or particularly interesting sphingolipid profiles are identified, for example in BY-2 cells. Equally importantly, these tools can be used in systems where growth and developmental phenotypes can most easily be studied, as discussed in the context of *P. patens*. Exciting topics that our field may be well positioned to investigate include the distribution and roles of the *ADS*- and *SFD*-like genes among plant lineages, the threshold level of GlcCers required for normal growth and development, the inferred functions of PLM proteins in sphingolipid metabolism, and the contributions of more extensively decorated GIPCs to plant biology.
